# Non-prescribing of clozapine for outpatients with schizophrenia in real-world settings: The clinicians’ perspectives

**DOI:** 10.1038/s41537-023-00423-3

**Published:** 2023-12-22

**Authors:** Michelle Iris Jakobsen, Stephen Fitzgerald Austin, Ole Jakob Storebø, Jimmi Nielsen, Erik Simonsen

**Affiliations:** 1 Department of Clinical Medicine, Faculty of Health and Medical Sciences, University of Copenhagen, Copenhagen, Denmark; 2 Psychiatric Research Unit East, Mental Health Services East, Region Zealand Psychiatry, Roskilde, Denmark; 3 The Psychiatric Research Unit, Region Zealand Psychiatry, Slagelse, Denmark; 4 Department of Psychology, Faculty of Health Science, University of Southern Denmark, Odense, Denmark; 5 Psychiatric Centre Glostrup, Unit for Complicated Schizophrenia, Mental Health Services in the Capital Region of Denmark, Copenhagen, Denmark

**Keywords:** Schizophrenia, Psychosis

## Abstract

Clozapine is the gold standard for treating treatment-resistant schizophrenia although continuously underutilized. Previous surveys of clinicians have found that some of the most frequently cited barriers to clozapine prescribing are related to the blood-monitoring requirements. However, these surveys tend to explore general perspectives and may not reflect the true impact of different barriers in real-world outpatient settings. This study aimed to explore this issue. First, by surveying the clinicians responsible for the treatment of 39 clozapine-eligible, yet clozapine-naive, outpatients with schizophrenia. Then, based on the survey results, explanatory interviews with the participating psychiatrists were conducted and analyzed thematically. The most frequently cited reason for non-prescribing of clozapine was the expected non-compliance with blood-monitoring requirements; however, overall stability and/or severe mental illness was chosen as the most important reason in most patient-cases. The qualitative analysis highlighted the combined impact of standard clinical practice, personal experiences, and organizational constraints on clozapine utility.

## Introduction

The atypical antipsychotic (AP) clozapine is the gold standard for treating treatment-resistant schizophrenia (TRS)^1,2^; however, it is continuously underutilized in most parts of the world^3^.

Previous studies have suggested that approximately half (44–52%) of all clozapine-eligible outpatients are clozapine-naive^4–6^ and that high-dose prescribing and/or antipsychotic polytherapy (APP) are much more frequently employed treatment strategies^4,7,8^ although not evidence-based nor guideline recommended—at least not until clozapine has been trialed.

Furthermore, clozapine treatment is usually initiated late in the treatment course, after several years of treatment resistance^9–12^ and several non-clozapine AP trials^13–15^, which seem to have a negative impact on the patient’s chance of response^16^.

The clinical reasons as to why clozapine is avoided might be quite relevant although rarely documented in case files^4,17^.

Previous surveys on clinicians’ perspectives on clozapine treatment have found that some of the most frequently mentioned barriers to clozapine prescribing are the patient’s refusal of treatment due to the need for frequent blood sampling or related adverse effects and the prescriber’s expectation of poor compliance and/or fear of severe adverse effects—adverse hematological effects in particular^18–22^.

Correspondingly, one of the most frequently mentioned clozapine-facilitating initiatives has been the implementation of point-of-care (POC) devices for capillary hematological monitoring^19–21,23^.

However, most previous studies are either based on clinicians’ general (i.e., not case-specific) perspectives on barriers to clozapine treatment, on in-patient populations, and/or on combined perspectives from a mixture of different clinical professions. Thus, the most frequently mentioned barriers and facilitators of clozapine prescribing might not reflect the barriers and facilitators of greatest impact on prescribing in real-world outpatient settings, which could help explain why clozapine utility has remained practically unchanged despite decades of research on the subject.

This comprehensive, two-phased, mixed-methods sequential explanatory design aimed to explore the case-specific clinician-perceived reasons for non-clozapine prescribing and corresponding facilitators of clozapine treatment for seemingly clozapine-eligible, yet clozapine-naive, outpatients with schizophrenia. We furthermore aimed to explore potential discrepancies between the most frequently chosen barriers/facilitators and the barriers/facilitators perceived to be the most important ones in each case as well as potential discrepancies between prescriber and non-prescriber perceptions.

## Results

### Phase one, Survey results

#### Participant characteristics and clinical ratings

**Fig. 1:**
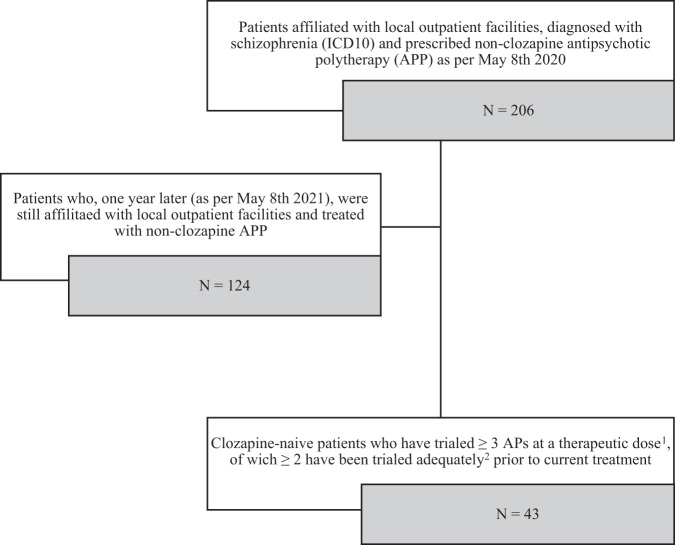
Screening for clozapine-eligible patients. The flowchart shows the process of screening for clozapine-eligible patients from a population of purposefully sampled outpatients with schizophrenia, treated with antipsychotic polytherapy (APP). ^1^ As defined in the manufacturer’s product labeling. ^2^ Therapeutic dose ≥ 6 weeks for oral prescriptions, ≥ 4 months for long acting injections.

Forty-three patients fulfilled our criteria for clozapine eligibility (Fig. 1).

In total, 70 questionnaires concerning 39 patients from three different centers were returned (response rate 83%). In some patient-cases, both the psychiatrist and the clinical care provider responsible for the patient’s treatment returned a questionnaire, in other patient-cases only one clinician returned a questionnaire.

The respondents consisted of 13 senior psychiatrists (all with prior experience in clozapine initiation) and 21 clinical care providers of which the majority (*n* = 17) were nurses. See Table 1 for more participant characteristics.

**Table 1:** Participant characteristics.

Participant variables	Descriptive statistics
Patients (*n*)	39
Males (*n* (%))	23 (59.0)
Age (years) (md (Q1–Q3))	38.0 (29.5–50.5)
APs^a^ trialed as regular prescriptions at a therapeutic^b^ dose (md (Q1-Q3))	5.0 (4.0-6.0)
APs^a^ trialed adequately^c^ (md (Q1-Q3))	4.0 (3.0–4.0)
Patients currently treated with AP^a^ as a long-acting injection (LAI) (*n* (%))	17 (43.6)
Patients with prior refusal of clozapine documented in case files (*n* (%))	7 (17.9)
Years since last documented refusal (md (Q1-Q3))	3.0 (1.5–7.5)
Psychiatrists (*n*)	13
Males (*n* (%))	4 (30.8)
Senior psychiatrists (*n* (%))	13 (100)
Care providers (*n*)	21
Males (*n* (%))	2 (9.5)
Nurses (*n* (%))	17 (81.0)
Other health-related training (*n* (%))	4 (19.5)

Notes:

Data is shown as numbers (n), numbers and proportions in percent (*n* (%)) or as medians and interquartile ranges (md (Q1–Q3)) as applicable.

^a^APs = antipsychotics.

^b^As defined by the manufacturer’s summary of product characteristics.

^c^Therapeutic dose for ≥ 6 weeks ( ≥ 4 months for APs prescribed as long-acting injections (LAI)).

As part of the survey, the clinicians were asked to rate the patients’ symptom severity (CGI-S), level of functioning (GAF-F), and treatment status (well-treated (i.e., adequately treated) or not).

The median clinician-rated CGI score of patients was 5.0 (interquartile range 5.0–6.3, full range 4.0–7.0) corresponding to the severity level “markedly ill”. Their median GAF score was 33.0 (interquartile range 25.0–42.1, full range 10–75), corresponding to the level “major impairment in several areas”. The coefficient of agreement between psychiatrists and care providers was 0.93 for CGI scores and 0.85 for GAF scores. See Table 2 on Clinical ratings.

**Table 2:** Clinical ratings in survey questionnaires.

Clinical ratings	In total	By psychiatrists (*n* = 13)	By care providers (*n* = 21)	Coefficient of clinician agreement	*P*-value
Patient cases rated (n)	39	33	37		
CGI^a^ Score (md (Q1-Q3))	5.0 (5.0-6.3)				
GAF^b^ Score (md (Q1-Q3))	33.0 (25.0-42.1)				
Cases with paired^c^ answers on CGI and GAF (n (%))	31 (79.5)				
CGI^a^ Score (md (Q1-Q3))		5.0 (5.0-7.0)	5.0 (5.0-6.0)	0.93^d^	0.52^e^
GAF^b^ Score (md (Q1-Q3))		25.5 (25.0-35.0)	35.0 (25.0-40.0)	0.85^d^	0.09^e^
Patients rated well-treated by ≥1 clinician (n (%))	27 (69.2)				
CGI^a^ Score (md (Q1-Q3))	5.0 (4.8-6.0)				
GAF^b^ Score (md (Q1-Q3))	31.8 (25.0-45.0)				
Patients not rated well-treated by any clinician (n (%))	12 (30.8)				
CGI^a^ Score (md (Q1-Q3))	5.8 (5.0-7.0)				0.08 ^f^
GAF^b^ Score (md (Q1-Q3))	34.0 (23.5-38.9)				0.64 ^f^
Cases with paired^c^ answers on treatment status^g^ (n (%))	29 (74.4)				
Patients rated well-treated (*n* (%))	20 (69.0)	18 (90.0)	9 (45.0)	0.35	< 0.0001 ^h^

Notes:

In the case-specific survey questionnaires, the clinicians were asked to rate the patients’ symptom severity (CGI score), functional impairment (GAF score), and treatment status (well-treated or not).

Data are shown as numbers (*n*), numbers, and proportions in percent (*n* (%)) or as medians and interquartile ranges (md (Q1–Q3)) as applicable.

^a^CGI Clinical Global Impression Scale.

^b^GAF Global Assessment of Functioning.

^c^Psychiatrist AND care provider answers regarding the same patient.

^d^Agreement at ±1 level of CGI or GAF.

^e^Differences between groups tested with the Wilcoxon signed-rank test.

^f^Comparison, Well-treated vs. not well-treated patients. Differences between groups tested with the Mann-Whitney U test.

^g^Treatment status well-treated/not well-treated.

^h^Differences between groups tested with the McNemar test.

In 27 cases (69.2% of cases), at least one clinician had rated the patient as well-treated. The psychiatrists rated the patients well-treated in 90% of cases and the care providers in 45% of cases (Table 2).

There were no significant differences between well-treated and not well-treated patients in terms of CGI or GAF scores (Table 2) nor in terms of the proportion of patients being treated with long-acting injections (LAI) of APs (well-treated LAI patients (*n* = 10 (37%)) vs. not well-treated LAI patients (*n* = 7 (58%)), *p* = 0.23, the “N-1” Chi-squared test).

#### Reasons for non-clozapine treatment

The distribution of chosen reasons (all chosen reasons as well as the chosen main reasons (i.e., the reason considered most important in each patient-case)) is shown in Table 3.

**Table 3:** Clinician-perceived reasons for current non-clozapine prescribing for included clozapine-eligible patients.

Reasons for non-clozapine treatment as listed in the survey questionnaires	1. Distribution of all chosen reasons (*n* (%))			2. Distribution of chosen main reasons (*n* (%))		
	In total (*n*_cases_=39)	By care providers (*n*_cases_=37)	By psychiatrists (*n*_cases_=33)	In total (*n*_cases_=38)	By care providers (*n*_cases_=36)	By psychiatrists (*n*_cases_=27)
1. Expected non-compliance with blood sampling	24 (61.5)*	19 (51.4)*	16 (48.5)*	6 (15.8)	6 (16.7)*	0 (0)
Expected patient refusal (any reason)	(24 (61.5))	(20 (54.1))	(11 (33.3))	(10 (26.3))	(8 (22.2))	(2 (7.4))
2. Due to blood sampling	18 (46.2),	16 (43.2),	8 (24.2),	5 (13.2),	5 (13.9),	0 (0),
3. Due to the side-effect profile	7 (18.0),	6 (16.2),	2 (6.1),	2 (5.3),	1 (2.8),	1 (3.7),
4. Due to other reasons	10 (25.6)	7 (18.9)	4 (12.1)	3 (7.9)	2 (5.6)	1 (3.7)
(Issues related to blood sampling in total) (i.e., expected non-compliance with OR refusal due to sampling)	(27 (69.2)*)	(24 (64.9)*)	(17 (51.5)*)	(11 (29.0))	(11 (30.6)*)	(0 (0))
5. Expected non-compliance with clozapine	19 (48.7)	14 (37.8)	14 (42.4)	9 (23.7)	6 (16.7)*	6 (22.2)
6. Other reasons	13 (33.3)	6 (16.2)	8 (24.2)	4 (10.5)	2 (5.6)	3 (11.1)
Added “other” reasons:						
- Cognitive impairment	8 (20.5),	4 (10.8),	5 (15.2),	1 (2.6),	0 (0),	1 (3.7),
- Substance abuse	6 (15.4),	2 (5.4),	5 (15.2),	1 (2.6),	0 (0),	1 (3.7),
- Old age	1 (2.6),	1 (2.7),	1 (3.0),	1 (2.6),	1 (2.8),	1 (3.7),
- Only temporary responsibility of treatment	1 (2.6),	0 (0),	1 (3.0),	1 (2.6),	0 (0),	1 (3.7),
- Clozapine is the treatment of last resort	1 (2.6)	1 (2.7)	0 (0)	1 (2.6)	1 (2.8)	0 (0)
7. Current treatment status	14 (35.9)	5 (13.5)	10 (30.3)	12 (31.6)*	3 (8.3)	11 (40.7)*
8. Don’t know	8 (20.5)	6 (16.2)	4 (12.1)	7 (18.4)	6 (16.7)*	3 (11.1)
Added reasons for not knowing:						
- Don’t know the patient that well	4 (10.3),	2 (5.4),	3 (9.1),	3 (7.9),	2 (5.6),	2 (7.4),
- Not responsible for drug treatment	4 (10.3)	4 (10.8)	0 (0)	4 (10.5)	4 (11.1)	0 (0)
9. Concerns about side effects	5 (12.8)	4 (10.8)	3 (9.1)	1 (2.6)	1 (2.8)	0 (0)
Added side effects:						
- Weight gain	2 (5.1),	2 (5.4),	1 (3.0),	1 (2.6),	1 (2.8),	0 (0),
- Sedation	2 (5.1),	2 (5.4),	0 (0),	0 (0),	0 (0),	0 (0),
- Patient concerned about side effects (unspecified)	2 (5.1)	1 (2.7)	2 (6.1)	1 (2.6)	1 (2.8)	0 (0)
10. Concerns about somatic issues	4 (10.3)	4 (10.8)	2 (6.1)	2 (5.3)	2 (5.6)	1 (3.7)
Added somatic concerns:						
- Current overweight	2 (5.1),	2 (5.4),	1 (3.0),	0 (0.0),	0 (0.0),	0 (0.0),
- Chronic obstructive pulmonary disease (COPD)	2 (5.1),	2 (5.4),	1 (3.0),	1 (2.6),	1 (2.8),	0 (0),
- Previous lymphoma	1 (2.6)	1 (2.7)	1 (3.0)	1 (2.6)	1 (2.8)	1 (3.7)
11. Due to a previous refusal of clozapine	3 (7.7)	2 (5.4)	3 (9.1)	1 (2.6)	1 (2.8)	1 (3.7)
12. Due to organizational issues	3 (7.7)	2 (5.4)	1 (3.0)	1 (2.6)	1 (2.8)	1 (0)
13. Due to treatment with contraindicated drugs	0 (0)	0 (0)	0 (0)	0 (0)	0 (0)	0 (0)

Notes

The table shows the distribution of case-specific reasons for non-clozapine treatment, chosen by clinicians. The clinicians were asked to choose all reasons of relevance to a specific patient-case (column 1), as well as the main reason (the reason considered the most important one in each patient-case) (column 2).

The distribution is shown as the number and percent (*n* (%)) of patient cases for which a particular reason is chosen; in total, by care providers and by psychiatrists.

^*^The most frequent choices are marked with an asterisk^*^.

In some patient-cases, both a psychiatrist and a clinical care provider returned a questionnaire regarding the same patient, however, in other cases only one clinician returned a questionnaire or one had skipped parts of the survey. The number of patient-cases with clinician responses thus differs between the total number of cases, the number of cases stratified by care providers and psychiatrists, and between choices and main choices.

The most cited reason for non-prescribing of clozapine, in total and stratified by psychiatrists and care providers, was the expected non-compliance with blood sampling (Table 3, column 1). When including the option “expected patient refusal due to blood sampling”, issues related to blood sampling were considered a barrier to clozapine prescribing in 69.2% of cases (Table 3, column 1).

When asked about the main reason preventing each patient from clozapine treatment (Table 3, column 2), the most frequent care provider answer remained related to blood sampling (in 30.6% of cases) whereas the psychiatrists more frequently rated the patient’s current treatment status as the main reason for non-clozapine prescribing (in 40.7% of cases). Issues related to blood sampling were not chosen as the main reason by any of the psychiatrists in any of the cases (Table 3, column 2). Concerns about compliance with drug treatment (i.e., with clozapine) was the second most chosen main reason among the psychiatrists (in 22.2% of cases)—four of these patients (67%) were treated with LAI APs as part of their APP.

#### Facilitators of clozapine treatment

The distribution of chosen facilitators (all relevant facilitators as well as the chosen main facilitators (i.e., the facilitator considered most relevant in each patient-case)) is shown in Table 4.

**Table 4:** Clinician-perceived facilitators of clozapine treatment for included clozapine-eligible patients.

Clozapine-facilitating initiatives listed in the survey questionnaires	1. Distribution of all chosen facilitators (*n* (%))			2. Distribution of chosen main facilitators (*n* (%))		
	In total (*n*_cases_=38)	By care providers (*n*_cases_=36)	By psychiatrists (*n*_cases_=31)	In total (*n*_cases_=37)	By care providers (*n*_cases_=35)	By psychiatrists (*n*_cases_=27)
1. Usage of topical anesthetics before blood sampling	1 (2.6)	1 (2.8)	0 (0)	0 (0)	0 (0)	0 (0)
2. Usage of point-of-care (POC) devices for finger-prick hematological monitoring	4 (10.5)	3 (8.3)	1 (3.2)	1 (2.7)	1 (2.9)	0 (0)
3. Decreased blood sampling frequency	12 (31.6)	10 (27.8)	3 (9.7)	5 (13.5)	5 (14.3)	0 (0)
4. Blood sampling and ECG recording performed in the out-patient clinic	2 (5.3)	1 (2.8)	1 (3.2)	0 (0)	0 (0)	0 (0)
5. Blood sampling and ECG recording performed in the patient’s home	12 (31.6)	9 (25.0)	5 (16.1)	9 (24.3)	7 (20.0)	2 (7.4)
(initiatives related to blood sampling (in total)) (i.e., usage of topical anesthetics OR POC devices OR blood sampling and ECG recording in clinic/at home OR decreased blood sampling frequency)	(16 (42.1))	(13 (36.1)*)	(7 (22.6))	(14 (37.8))	(13 (37.1)*)	(2 (7.4))
6. Special-developed patient information on clozapine treatment (e.g., videos with specialists and patients sharing their own experiences with clozapine)	4 (10.5)	4 (11.1)	0 (0)	1 (2.7)	1 (2.9)	0 (0)
7. Employment of peers/clozapine ambassadors	2 (5.3)	2 (5.6)	0 (0)	1 (2.7)	1 (2.9)	0 (0)
8. Reference to a specialized clozapine unit for clozapine initiation	5 (13.2)	5 (13.9)	1 (3.2)	1 (2.7)	1 (2.9)	0 (0)
9. Access to other administration forms of clozapine (e.g., as intra-muscular injections (not long-acting))	3 (7.9)	3 (8.3)	0 (0)	2 (5.4)	2 (5.7)	0 (0)
10. Enforced commencement	6 (15.8)	5 (13.9)	3 (9.7)	5 (13.5)	2 (5.7)	3 (11.1)
11. Other initiatives	21 (55.3)	16 (44.4)*	12 (38.7)	2 (5.4)	1 (2.9)	1 (3.7)
Added initiatives:						
- Long-term admission/residency at a psychiatric institution/close follow-up	14 (36.8),	11 (30.6),	9 (29.0),	2 (5.4),	1 (2.9),	1 (3.7),
- Quit substance abuse	7 (18.4),	3 (8.3),	4 (12.9),	0 (0),	0 (0),	0 (0),
- Weight loss	2 (5.3),	1 (2.8),	1 (3.2),	0 (0),	0 (0),	0 (0),
- Clozapine available as a long-acting injectable	1 (2.6),	1 (2.8),	0 (0),	0 (0),	0 (0),	0 (0),
- Possibility to refrain from blood sampling	4 (10.5)	3 (8.3)	1 (3.2)	0 (0)	0 (0)	0 (0)
12. No initiatives are relevant for this patient	28 (73.7)*	13 (36.1)*	23 (74.2)*	28 (75.7)*	9 (25.7)*	19 (70.4)*
13. Don’t know	5 (13.2)	5 (13.9)	2 (6.5)	7 (18.9)	5 (14.3)	2 (7.4)

Notes

The table shows the distribution of case-specific facilitators of clozapine treatment, chosen by clinicians. The clinicians were asked to choose all facilitators of relevance to a specific case (column 1), as well as the main facilitator (the facilitator considered most relevant in each patient-case) (column 2). The distribution is shown as the number and percent (n (%)) of patient-cases for which a particular facilitator is chosen.

^*^The most frequent choices are marked with an asterisk^*^.

In some patient-cases, both a psychiatrist and a clinical care provider returned a questionnaire regarding the same patient, however, in other cases only one clinician returned a questionnaire or one had skipped parts of the survey. The number of patient-cases with clinician responses thus differs between the total number of cases and the number of cases stratified by care providers and psychiatrists.

When the clinicians were asked to choose between proposed initiatives that might facilitate clozapine treatment for the patient in question, the most frequently chosen answer was “No initiatives are relevant for this patient” (in 73.7% of cases) (Table 4, column 1).

When the clinicians were asked to choose a main answer in terms of case-specific clozapine facilitators (Table 4, column 2), the “No initiatives are relevant for this patient” answer continued to be the most frequently chosen one, both in total and amongst the psychiatrists (in 75.7% and 70.4% of cases, respectively). The care providers, however, seemed to be more optimistic about the initiatives aiming at blood-monitoring issues (in 37.1% of cases) (Table 4, column 2).

The answer “No initiatives are relevant for this patient” was often (in 61.1% of the answers) accompanied by a qualitative, free text note stating that the patient was either well-treated (in 59.1% of answers), as well-treated as possible (in 18.2% of answers) and/or too ill for clozapine treatment (in 59.1% of answers).

### Phase two, Interview results

#### Selected focus areas for interviews

The survey results suggested that most patients were at least markedly ill and of major functional impairment. However, the psychiatrists considered the patients well- (i.e., adequately) treated in most cases. Being well-treated was also the psychiatrists’ most frequently chosen main reason (i.e., most important reason) as to why patients were not treated with clozapine. In contrast, the care providers most frequently chose blood sampling as their main reason for non-clozapine treatment and facilitators of blood sampling as the main facilitator. The psychiatrists did not consider initiatives aiming at blood sampling, or any other initiatives, to be important facilitators of clozapine treatment. The reasons why, given as supplementary, qualitative notes, were that the patients were either well-treated, as well-treated as possible, or too ill for clozapine treatment.

Based on these results, several questions emerged, warranting further elaboration:

1. When is a patient considered eligible for a trial of clozapine?
2. What does it mean when a patient is considered “too ill” for clozapine treatment?
3. What does it mean when a patient is considered markedly/severely ill but “too well-treated” for clozapine treatment?
4. How do the psychiatrists respond to the survey results suggesting that psychiatrists, in contrast to care providers, do not consider blood sampling to be a major barrier to clozapine prescribing and that interventions aiming at blood-sampling issues are of only minor importance?

These four questions became the focus areas of the subsequent explanatory interviews.

#### Material and participants

Ten psychiatrists (3 males, 7 females), representing all three centers, agreed to be interviewed and audio recorded. Interviews ranged from 26.3 to 59.1 min in duration.

### Focus area 1. Elaborations on clozapine eligibility

**Fig. 2:**
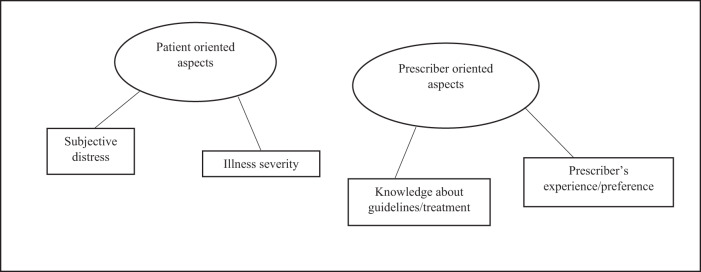
Thematic map on “Clozapine eligibility”. The map shows the two major themes identified on clozapine eligibility based on the explanatory interviews with participating psychiatrists. Within each theme, two subthemes were identified.

The psychiatrists’ perceptions of clozapine eligibility were reflected in two major themes; (1) The patient-oriented aspects and (2) The prescriber-oriented aspects (Fig. 2):

1. Patient-oriented aspects: All psychiatrists defined a clozapine candidate as a patient who was at least markedly ill (CGI 5 ≥) and of low functioning ( <GAF 50). Half of the psychiatrists further stated that the patient in addition should be subjectively distressed due to their illness:

“*and… if the patients are distressed by their symptoms*” (TRP04).

2. Prescriber-oriented aspects: The vast majority of psychiatrists knew that clozapine was a third-line treatment, but all psychiatrists stated that clozapine usually was initiated far later than that:

*“After a minimum of 3 antipsychotics. Minimum. Usually, more will be trialed…4 or 5”* (TRP02).

Some psychiatrists thought of it as a drug of “last resort” and stated that they deliberately postponed the initiation of clozapine therapy because they wanted to first trial APs that could be given as a long-acting injection (LAI), or were considered less of a burden to the patients in terms of side effects and monitoring requirements:

*“To me, clozapine is not a 3rd line treatment…it’s later in the treatment course. I have great success with initiating patients in treatment with depot medications, so…to me, that comes first”* (TRP01).

*“I think you should use clozapine when you have trialed all other antipsychotics (…) there are side effects to it, and…lots of blood tests (…) clozapine is like the last drug. Why should you trial the last choice as one of the first?”* (TRP06).

All psychiatrists stated that they considered clozapine an effective AP, but not all thought it more effective than other APs:

*“I wouldn’t say it’s better than any other antipsychotic…it’s not my clinical experience no”* (TRP03).

Psychiatrists who stated that they deliberately postponed clozapine were all among the psychiatrists who had experienced clozapine as unsuperior to other APs.

### Focus area 2. Elaborations on being too ill for clozapine treatment

**Fig. 3:**
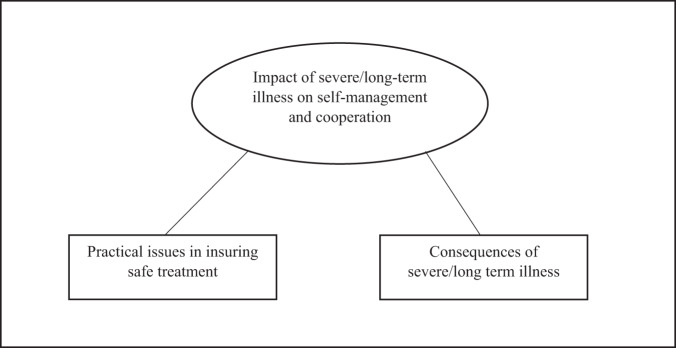
Thematic map on “What does it mean to be too ill for clozapine treatment?”. The map shows the major theme identified when the participating psychiatrists elaborated on this focus area. The theme was constructed on the basis of two sub-themes.

Being too ill for clozapine treatment was reflected in one major theme; The impact of severe/long-term illness on self-management and cooperation (Fig. 3):

A patient who was too ill for clozapine treatment was described as severely ill and/or cognitively disabled due to their illness and therefore unable to understand and cooperate with all aspects of clozapine treatment:

*“Cooperation needs to be possible (…) there’s a lot they should be able to do.”* (TRP08).

Especially noncompliance with medications was a concern:

*“The most severely ill patients, they cannot manage to take their pills on their own (…) of course, some patients could be assisted, but in practice, it’s difficult to make clozapine treatment work with these patients”* (TRP01).

Some psychiatrists mentioned the perceived lack of professionalism in the patient’s network, as well as limited access to monitoring in the patients’ homes, as a safety and feasibility concern:

*“The organizational framework doesn’t allow us to provide the patients with a proper medical treatment”* (TRP10).

Depending on the duration of severe illness, these patients were furthermore considered a somewhat “lost cause” beyond clozapine treatment:

*“Professionally, you know, over time…the prognosis is poor (…) There isn’t much more to gain. Then it’s also your job, as a doctor, to be realistic”* (TRP10).

### Focus area 3. Elaborations on being too well-treated for clozapine treatment

**Fig. 4:**
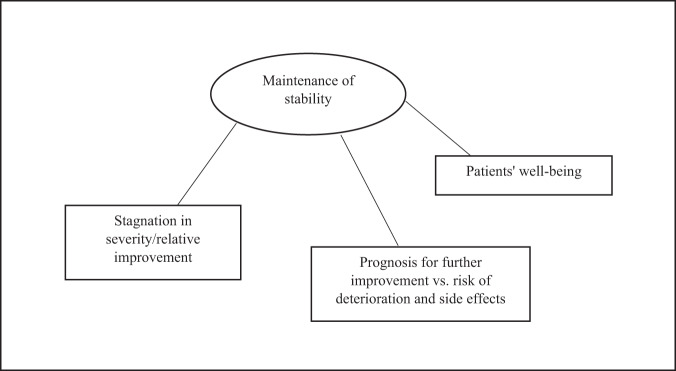
Thematic map on “What does it mean to be too well treated for clozapine treatment though ≥ markedly ill?”. The map shows the main theme and three embedded subthemes identified as the interviewed psychiatrists elaborated on this focus area.

Being too well-treated for clozapine treatment in the context of marked or severe illness was captured within one major theme; Maintenance of stability (Fig. 4):

A patient who was considered well-treated or as well-treated as possible though markedly or severely ill was by all psychiatrists defined as a patient who had reached “stability” in their illness and who had improved enough or at least as much as possible. As one psychiatrist stated:

*“So, for example, we think, okay, it doesn’t get worse, so it’s “stably unwell”. But it’s good for that person (…) sometimes, even if the patient wishes it, we reckon that it doesn’t get any better than this”* (TRP02).

Another one stated that:

*“*(…) *not necessarily that they are symptom-free, but that they have reached some kind of stability, where they, for example, experience quality of life… can sleep at night and enjoy themselves to some extent”* (TRP05).

The patient’s well-being was generally important to the psychiatrists when considering clozapine and the vast majority of psychiatrists mentioned the absence of subjective distress as a marker of being well-treated:

*“In general, patients are well-treated when they are no longer distressed (due to their illness)”* (TRP03).

Some psychiatrists further explained that they, or the patients themselves, often were reluctant to change the medications in the case of stability, in fear of subsequent deterioration or unnecessary side effects without the prospect of a significant improvement in symptom severity:

*“I’m not sure that I will gain anything from it (switching to clozapine), other than a sedated, drooling patient (…) there is also a lot to lose, you know. It doesn’t necessarily go extremely well”* (TRP05).

### Focus area 4. Psychiatrists’ perspectives on the survey results

#### 4.a. Blood sampling as a barrier and/or facilitator of clozapine prescribing

**Fig. 5:**
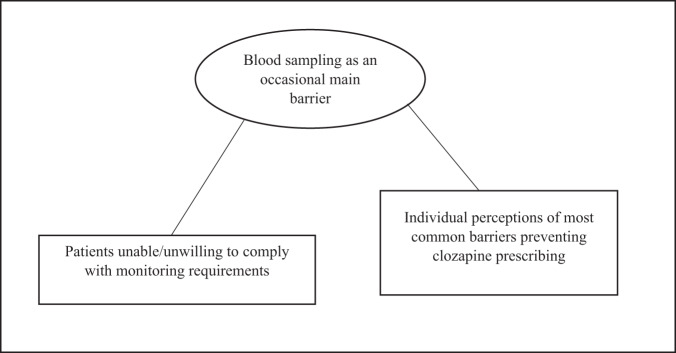
Thematic map on “Blood sampling as a barrier to clozapine prescribing”. The map shows the psychiatrists’ perspectives on blood sampling as a barrier to clozapine prescribing. The figure shows one overarching theme, “Blood sampling as an occasional barrier” and two subthemes.

When exploring the psychiatrists’ perspectives on blood sampling in relation to clozapine prescribing, one overarching theme was identified: Blood sampling as the occasional main barrier and facilitator (Fig. 5).

Several psychiatrists spontaneously recalled patients whom they considered eligible for clozapine therapy, but to whom they could not prescribe it due to the patients’ unwillingness or inability to have blood drawn regularly. However, the majority of psychiatrists confirmed the survey results suggesting that blood sampling is not the usual main barrier:

*“There are some patients, where I have refrained from clozapine because… I know that this person will never have a blood test taken. Like never. They just won’t. But, it’s very few (patients)”* (TRP09).

When blood sampling was mentioned as a barrier, the concern was solely related to compliance with the guideline-defined frequency of sampling. Adverse hematological effects, on the other hand, seemed to be a minor concern to the psychiatrists:

*“No, it’s not really a concern (adverse hematological effects) (…) I don’t think I’ve ever seen a clozapine-induced neutropenia. But I’ve seen it with other antipsychotics”* (TRP10).

Which barrier each psychiatrist perceived to be the most common one preventing them from clozapine prescribing differed substantially. Some psychiatrists stated that it was the risk of cardiac toxicity combined with the expected non-compliance with ECG monitoring and difficulties in obtaining home ECG recordings. Others stated that it was the expected non-compliance with clozapine itself and/or the subsequent re-titration regimen, the patient’s cognitive deficit and lack of understanding of the clozapine treatment in general, the burden of adverse effects (weight gain in particular) and/or burden of frequent blood samplings, stability in the patient’s illness or the prescribers’ sparse experience with clozapine treatment.

The major concerns seemed to be loss of drug compliance, expected difficulties in obtaining ECG recordings, or the patient’s current overweight.

Most psychiatrists acknowledged during the interview that POC devices for finger-prick blood sampling would make hematological monitoring easier on both patients and clinicians and that it in some cases probably could facilitate clozapine treatment:

“*Well, in some cases, if the patient is afraid of needles, they might be persuaded to a tiny prick on the finger (…) yes, I think, in some cases, it could definitely make a difference”* (TRP05).

However, all psychiatrists agreed that capillary monitoring alone, or other isolated interventions to facilitate blood sampling, would not dramatically increase clozapine prescribing:

“*No, I think that, for most patients, it doesn’t matter, because blood sampling is not the problem*” (TRP01).

To increase clozapine utility several simultaneous changes were perceived necessary: e.g., educational initiatives, political prioritizing and audit on clozapine treatment, more liberal blood monitoring requirements, longer admissions and/or easy access to home-ECG recordings, aided drug-compliance and weight reduction, and more time per outpatient for follow-up and management of side effects.

#### 4.b. Differences in the psychiatrists’ and care providers’ perspectives on barriers and facilitators of clozapine treatment

**Fig. 6:**
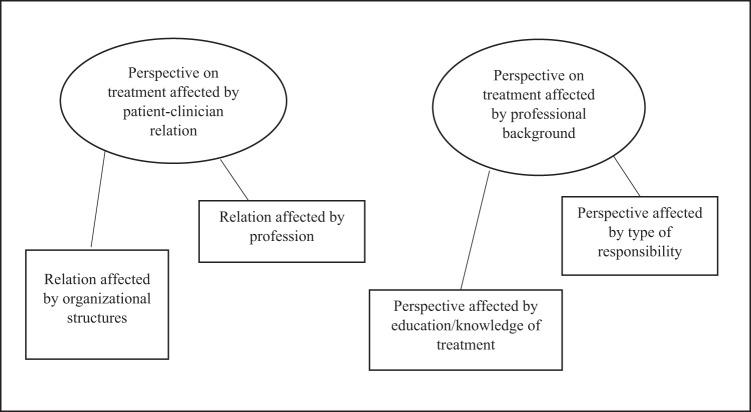
Thematic map on “Differences in the psychiatrists’ and care providers’ perspectives on barriers and facilitators of clozapine treatment”. The map shows the two identified themes and embedded subthemes, as expressed by the interviewed psychiatrists.

When asked about the differences in psychiatrists’ and care providers’ weighting of barriers and facilitators, two themes were identified: 1) Perspective on treatment affected by patient-clinician relation and 2) Perspective on treatment affected by professional background (Fig. 6).

1. Perspective on treatment affected by patient-clinician relation: Most psychiatrists answered that the care providers probably knew the patients and their challenges better than the psychiatrists did, due to the differences in the number and form of contacts:
  *“They are closer to the patients (…) when you have, like, 240 patients you uh…we don’t know the patients in the same way the care providers do. The care provider might have come to the patient’s home and… they might have talked about children and food and…you know, everyday stuff. Then it’s perhaps easier to say things”* (TRP04).
  Being a doctor was furthermore experienced as an obstacle, in some cases, to finding out how the patient was doing:
  *“It is not uncommon that the doctor gets a different (more positive) answer than the nurse does”* (TRP04).
2. Perspective on treatment affected by professional background: Some psychiatrists pointed out that the care providers in addition were positioned differently in terms of professional standpoint and responsibility:

*“Doctors might be more prognostic”* (TRP01), or

“*Doctors are realistic, basically. After all, it’s the doctors who have the expertise*” (TRP10),

“*I think that one of the reasons is that… it’s the doctor’s responsibility what happens to the patient*”(TRP09).

## Discussion

### Integration of quantitative and qualitative results and discussion of main results

This study investigated the clinician-perceived reasons for current non-clozapine prescribing for 39 clozapine-naive outpatients with schizophrenia. All patients were seemingly eligible for a trial of clozapine according to guidelines (≥moderate symptom severity and/or ≥ moderate functional impairment as assessed by their treating clinicians, responding inadequately to AP monotherapy ≥ 12 weeks (we considered continued or recurrent APP treatment ≥ 1 year as a pseudo measure for this), trialed ≥ 2 APs adequately)^2^. Quantitatively, we found that issues related to blood sampling, expected non-compliance with blood sampling in particular, was the most frequent clinician-perceived reason for non-clozapine treatment in both psychiatrist and care provider questionnaires. These results are in line with those of previous studies reporting that some of the most frequently clinician-cited barriers to clozapine prescribing are related to blood sampling^18–21^ and that the general perspectives on clozapine treatment largely correspond between prescribers and non-prescribers^24^.

However, an interesting shift in the weighting of reasons appeared, when the clinicians were asked to choose a main reason. The care providers hold on to their perception of blood sampling being a barrier and chose this as the main obstacle to (and facilitator of) clozapine treatment in most cases. The psychiatrists (the prescribers themselves), on the other hand, had another focus in most cases: the patient’s current treatment status. Explained qualitatively, this referred to a consideration of the prognostic aspects of changing medications vs. overall stability on current APP treatment and/or the patient’s inability to safely manage all aspects of their own treatment. It furthermore meant that no single interventions were considered adequate facilitators of clozapine treatment.

The psychiatrists’ reasons for focusing on stability and prognostic aspects of changing medicines were, in all cases, anchored in personal clinical experiences with clozapine treatment-burden, -efficacy, and organizational constraints. The psychiatrists were genuinely concerned about the patients’ safety and well-being if switched to clozapine, which resulted in the active postponement or avoidance of clozapine treatment in order to protect the patients.

In this light, the psychiatrists tended to accept quite high levels of symptom severity and quite low levels of functioning as patients being “stably ill” (and therefore too well-treated for clozapine) and/or “beyond clozapine treatment”.

They further expressed the employment of real-world clozapine eligibility criteria far more restrictive, and to some point contradictive, than those suggested by current guidelines: eligible patients should be subjectively distressed, at least marked ill (CGI ≥ 5), of low functioning (GAF ≤ 50), and they should have trialed several other APs ( ≥ 3), preferably in different formulations, before clozapine was to be considered. At the same time, the patients should not have been too ill for too long, and neither should they be too ill and/or too low functioning to manage treatment safely on their own.

Consequently, the employment of such altered clozapine-eligibility criteria entails a clinical practice in which the number of clozapine-eligible patients, as well as the timeliness of clozapine-eligibility, is negatively affected.

If stably-, yet markedly or severely, ill patients are considered better off without clozapine treatment, or even beyond clozapine treatment, and therefore not offered clozapine, they might also be considered treatment-denied as argued elsewhere^25^.

Furthermore, clozapine response rates seem affected by the patient’s age, duration of illness, and number of APs trialed prior to clozapine initiation^16^. Thus, when clozapine is introduced late in the treatment course, or even as a “last resort” treatment, the psychiatrists unintendedly end up confirming their own negative experiences with clozapine. At this point, the chances of response are in decline with consequences to the patients’ long-term prognosis, connections to job/study/social network, etc. In that sense, “protecting” the patients from clozapine treatment for as long as possible, unfortunately ends up depriving the patients of a timely trial of clozapine, and therefore it risks depriving them of their chances of further improvement.

However, none of the psychiatrists seemed aware of this connection, which stresses the need for continuous and targeted training for prescribers.

Another important aspect in terms of knowledge was the perception that the patient’s overweight was a contraindication for clozapine therapy. Previous research has shown that patients who have gained weight on previous treatment experience none or only minor weight gain when switched to clozapine^26^. Current overweight should therefore not be considered a legitimate reason for treatment stagnation. Side effects were, however, mostly a concern amongst the care providers. So were concerns about somatic issues and the answers “Don’t know” and “I’m not responsible for drug treatment” when asked about reasons for non-clozapine treatment. According to the psychiatrists, the care providers know the patients better and they have more frequent contact with the patients. The care providers could therefore be an important resource in terms of assessing and drawing attention to clozapine eligibility as well as in handling individually adapted treatment and/or monitoring programs which stresses the importance of clozapine knowledge-dissemination and training of the clinical care providers as well.

To date, no published studies have asked the clozapine-eligible, yet clozapine-naive, outpatients themselves about their willingness to trial clozapine, the reasons why, and which initiatives (if any) might facilitate their willingness. Future studies in this area would help clarify the importance of illness stability vs. the possibility of improvement and thereby the relevance of the concerns expressed by the clinicians.

### Strengths and limitations

With this comprehensive investigation of case-specific clinician-perceived barriers and facilitators of clozapine prescribing, we provide evidence suggesting that blood sampling is not the most important barrier to clozapine prescribing in most cases and that the implementation of POC devices might have less of an impact on clozapine utility than previously assumed if not concurrently combined with other types of interventions.

Furthermore, our results suggest that stability (even low-functioning stability) is prioritized over switching to clozapine and that some patients are considered too ill for clozapine treatment.

These results contradict the general assumptions about barriers to clozapine prescribing and that clozapine is for the most severely ill patients.

Our study thus offers a different take on the real-world reasons for clozapine underutilization and the required solutions thereof—a take that we consider of major importance to future implementation strategies.

Limitations to this study include the small sample sizes and local study set-up which constraints the power of statistical testing as well as the overall generalizability.

However, the distribution of clinician-perceived barriers to clozapine prescribing in our study (when choosing any barrier of relevance to a case) showed similar to those in previous studies on general/non-case-specific perspectives on barriers^18–21^ which suggests some level of generalizability in terms of perceived main barriers and facilitators as well.

Treatment resistance affects approximately 1/3 of all patients with schizophrenia and the number of eligible participants for a study on TRS patients will therefore, as per definition, be limited. A study of TRS patients not yet treated with clozapine will naturally relate to an even smaller number of patients. Furthermore, certain guideline-defined criteria must be met for a patient to be deemed TRS^2^ and therefore eligible for clozapine treatment. However, real-world clinical practice is not always guideline-adherent^4,9,27,28^. In recognition of that, we chose a study design that would reduce the importance of sample sizes: The mixed-methods sequential explanatory design. Within this design, quantitative survey data integrates with the subjective elaborations and context obtained from qualitative data, thereby generating a much deeper and more authentic understanding of a given topic than any of the methods (survey vs interviews) would generate on their own.

In our study, clozapine eligibility was assessed based on current AP prescriptions and retrospective case notes, entailing the risk of including a number of “false positive” clozapine-eligible patients. As a means to reduce that risk, we added the criterion of three trialed APs at an effective dose. This addition further had the advantage that we could ask the clinicians why the patient had not commenced clozapine treatment yet, instead of simply pointing out that the patient now should be considered for clozapine treatment.

One year of recurrent or continued APP treatment was one of the inclusion criteria for a patient to be considered clozapine eligible, which is far longer than the minimum of 12 weeks required in the TRRIP consensus guideline. One year was selected for the following three reasons: (1) The Covid pandemic, which postponed the initiation of the survey phase from the autumn of 2020 to the spring of 2021. (2) Reduced/altered contacts between patients and clinicians during the lockdown periods of the pandemic, which meant that suboptimal treatments might have gone unnoticed for longer than usual. (3) New patient/clinician pairs due to major local organizational changes in the spring of 2021. The objective of this study was to explore the clinicians’ perceived reasons for non-clozapine prescribing. To that end, we deemed it in the study’s interest to wait and let the clinicians have a fair chance to see the patients and consider the need for clozapine treatment (or the lack thereof) before starting the survey. The true number of eligible patients may therefore have been higher than depicted in this study; however, the strength of this study lies within the case-specific and therefore resource-demanding study design, which provides a glimpse of real-world practice that is rarely seen in this area of research.

### Clinical implications

Real-world clozapine eligibility criteria seem to differ from guideline criteria, which, in practice, reduces the number of clozapine-eligible patients as well as the timeliness of clozapine commencements. Adherence to treatment guidelines with a timely trial of clozapine should be strived for, especially in the early stages of psychosis treatment, to avoid treatment stagnation on suboptimal response levels by premature APP prescribing and delayed clozapine prescribing.

Blood sampling was not the main psychiatrist-perceived barrier to clozapine prescribing and the implementation of POC devices for hematological monitoring was not perceived to be an important enabler of clozapine prescribing in most cases. Thus, initiatives aiming at facilitating blood sampling may have less of an impact on clozapine prescribing than previously assumed, if not concurrently combined with other initiatives.

Knowledge deficiencies regarding clozapine treatment were observed amongst both prescribers and non-prescribers, as were organizational constraints affecting the perceived safety of clozapine prescribing for outpatients. To address both of these issues, organizations should ensure that their clinicians (care providers as well as prescribers) are adequately trained in providing clozapine treatment so that clozapine eligibility is recognized in a timely manner and treatment offered promptly. Previous evidence^29^ has shown that targeted training has the potential to increase clozapine prescription rates substantially in outpatient settings. Organizations should furthermore structure treatment facilities in terms of equipment and distribution of tasks to ensure that patients in need of clozapine treatment can receive it in practice. A way to promote this could be by implementing e.g., POC devices for both hematological and cardiac monitoring and distributing some of the responsibilities with clozapine-monitoring (e.g., blood sampling, ECG recordings, screening for side effects), to the care providers. Such interventions could make the prescribers more prone to give clozapine a chance.

## Conclusion

Blood sampling was the most frequently chosen barrier to clozapine prescribing by both psychiatrists and care providers, however, the psychiatrists did not perceive it to be the most important barrier. Instead, clozapine prescribing seems avoided for otherwise eligible outpatients with schizophrenia if the patients are too severely mentally ill to manage all aspects of clozapine treatment on their own, or if they have reached overall stability on non-clozapine APP treatment –regardless of illness severity.

Prescribers need to be aware that stabilizing the patients on APP treatment, before clozapine has been trialed, risks stagnating treatment on a suboptimal response, which reduces their chances of ever being offered clozapine and ultimately deprives them of that chance of further improvement they might could have had with a timely trial of clozapine.

Concurrently, organizations should be aware that they are responsible for the feasibility of guideline-adherent treatment and they should act accordingly.

This study emphasizes that the key to a successful increase in clozapine utility is multifactorial and that both prescriber and organizational issues have to be addressed at the same time.

Future studies examining clozapine-naive outpatients’ perspectives on switching to clozapine might also help guide the relevance of different implementation strategies.

## Methods

A mixed-methods sequential explanatory design was employed, in which the study was conducted in two sequential phases, building upon each other. First, an initial survey phase generated quantitative survey results, which then informed the formation of a subsequent interview phase generating qualitative results. The intent of the qualitative follow-up phase was to have selected respondents explain or elaborate on the quantitative results within a context, thus providing a more in-depth understanding of the topic^30^.

### Phase 1 (survey)

#### Material and participants

Electronic medical records of 206 purposefully sampled outpatients with a diagnosis of schizophrenia (ICD10) and a prescription of non-clozapine antipsychotic polytherapy (APP) were screened for clozapine-eligible patients.

The 206 APP patients were identified on the 8th of May, 2020, as part of a local quality assessment project, reported upon elsewhere^4^.

Patients were considered likely to be clozapine eligible if they, one year later (as of the 8th of May, 2021), were:

- still affiliated with one of our three outpatient facilities,
- had a prescription of non-clozapine APP (indicating a recurrent or continued insufficient response to treatment with APs in monotherapy), had trialed ≥ 3 different non-clozapine APs at a therapeutic dose (as defined in the manufacturer’s product labeling), of which
- ≥ 2 APs had been trialed adequately (at a therapeutic dose for at least 6 weeks/4 months if prescribed as a long-acting injectable) prior to current treatment, excluding treatment periods described with questionable compliance,
- were clozapine naive

Data regarding current and former AP treatment, demographics, and local outpatient affiliations were retrieved by the lead investigator (author MIJ).

Following the identification of eligible patients, the team of clinicians responsible for their treatment (both the psychiatrists (TRPs) and the primary clinical care providers (CCPs)) were contacted and asked to fill in patient-specific questionnaires. The construction of survey questionnaires was based on the current literature and discussions with specialists and external clinical psychiatrists. English versions of the survey questionnaires are available as supplementary material (S1). The survey included assessments of symptom severity (using the Clinical Global Impression Scale (CGI-S)), level of functioning (using the Global Assessment of Functioning (GAF-F)), current treatment status (is the patient well-treated/adequately treated?), reasons for non-clozapine treatment and initiatives that might facilitate clozapine treatment for the patient in question. The questionnaires provided 13 pre-defined choices of reasons and facilitators respectively, including a blank “Other” option and a comment area to fill in as needed, giving the clinicians the opportunity to provide further choices and explanations. Finally, the clinicians were asked to state which of the chosen reasons and facilitators they considered the most important/relevant one in the particular case.

Data collection went on from May 2021 until October 2022.

#### Quantitative analyses

Data on participant characteristics and survey responses were analyzed with descriptive statistics using Excel (version 2013) and the statistical software R^31^ and RStudio^32^. Treatment status was assessed as a nominal outcome with four possible answers: “yes”, “no”, “don’t know” and “other”. Extra lines were provided for elaboration. Depending on this elaboration, assessments on treatment status were collated to form a dichotomous variable: well-treated and not well-treated.

Distributions of normality were tested visually with histograms and Q-Q plots. Differences between groups (e.g., psychiatrist vs. care-provider assessments or well-treated vs. not well-treated patients) were tested as applicable, using the non-parametric Wilcoxon signed-rank test or the McNemar test for paired data and the Mann-Whitney U test or the “N-1” Chi-squared test for unpaired data. All *p* values were 2-tailed at the significance level of <0.05.

### Phase 2 (interviews)

#### Material and participants

Following the analysis of survey responses, the authors discussed the findings and selected relevant focus areas warranting further elaboration. An interview guide was subsequently developed, based on the survey results. An English version of the interview guide is available as supplementary material (S2).

Explanatory interviews with the psychiatrists who had participated in the survey were conducted in December 2022 and January 2023.

Interviews were semi-structured and the psychiatrists were initially encouraged to elaborate on their experiences and perspectives on clozapine treatment in relation to eligibility, utility, and efficacy. They were furthermore asked to define a CGI-S and GAF-F of a standard patient considered eligible for clozapine treatment and to comment on the main findings from the survey, presented by the interviewer (author MIJ). Field notes were generated during and/or immediately after each interview. All interviews were audio-recorded and later transcribed verbatim by an experienced research assistant.

#### Qualitative analyses

Interviews were analyzed thematically at the semantic level in accordance with the framework developed by Braun & Clarke^33^. The qualitative data analysis software NVivo (v.12)^34^ was used to organize and manage the data during analysis.

First, field notes and transcripts were read through in their entire length to obtain an overall understanding of the data.

Then, the transcripts were coded deductively according to focus area, and the text collated accordingly. Collated text on each focus area was subsequently coded inductively and organized into preliminary themes and subthemes. The preliminary analysis was conducted by the interviewer (author MIJ) and then reviewed by an experienced qualitative researcher (author SFA) to promote trustworthiness by ensuring that themes were grounded within the data. The formation of themes and subthemes was further adapted in an iterative process.

#### Ethics and permissions

According to Danish law, the project does not need ethical approval.

The Regional Data Agency approved the use of study data (ID: REG-092-2020) and the Regional legal authorities permitted the retrieval of data from medical records and for the clinicians to answer the patient-specific questionnaires (ID: R-21013123). Clinicians were considered consenting with survey participation if returning a filled-in questionnaire and with interview participation if providing written informed consent.

All data from medical records, survey responses, and interviews (sound files and transcripts) has been anonymized.

### Supplementary information

S1

S2

## Data Availability

The data that support the findings of this study are not openly available due to reasons of sensitivity. Data are, however, available from the authors upon reasonable request and with permission from the Mental Health Services East of Region Zealand Psychiatry.
