# Supplementary material for: Non-prescribing of clozapine for outpatients with schizophrenia in real-world settings: The clinicians’ perspectives
Source: Schizophrenia (Heidelb). 2023 Dec 22;9(1):91. doi: 10.1038/s41537-023-00423-3 (PMC10746712; doi:10.1038/s41537-023-00423-3)
Supplement: Supplementary file 1 — S1 [file 41537_2023_423_MOESM1_ESM.pdf]

Questionnaire concerning clinical barriers and facilitators for starting clozapine treatment in patients with schizophrenia

Based on a journal audit, it appeared that this patient (hereafter referred to as X), according to his medical history, met the criteria for treatment with clozapine. X had, at the time of the audit, not yet been treated with clozapine. There may be several reasons why X has not tried clozapine treatment, and with this questionnaire, we wish to uncover the reasons for that, as well as the needed facilitators if clozapine were to become relevant for X.

Background data:

1) What is your biological sex?

☐ Male ☐ Woman

2) What kind of clinician are you?

☐ Doctor ☐ Physiotherapist ☐ Nurse ☐ Social and health assistant ☐ Other____________________________

For doctors:

☐ Intern ☐ Resident ☐ Psychiatrist ☐ Senior psychiatrist

3) Have you ever commenced patients in clozapine therapy?

☐ Yes ☐ No

4) For how long have you treated this patient?

☐ 0-3 months ☐ 3-6 months ☐ 6-12 months ☐ 12+ months

5) How many times have you met this patient?

☐ 0 times ☐ 1 time ☐ 2-3 times ☐ 4-6 times ☐ 6+

6) How often do you have (any) contact with this patient?

☐ Daily ☐ Several times a week ☐ Weekly ☐ Several times monthly ☐ Monthly ☐ Quarterly ☐ Half-yearly

☐Annually ☐ Other____

7) Based on your latest knowledge of this patient, where on the Clinical Global Impression Scale (CGI-S) would you assess the severity of the patient's illness?

Score	Clinical Global Impression (illness degree)	Place x
1	No signs of mental illness	
2	Doubtful as to whether a mental illness exists	
3	Mild degree of illness	
4	Moderate degree of illness	
5	Severe degree of illness	
6	Very severe degree of illness	
7	Amongst the most seriously ill patients within the psychiatric diagnosis to which the patient belongs	

8) Based on your latest knowledge of this patient, where on the Global Assessment of Functioning (GAF-F) scale would you assess the patient to be?

Score	Symptom level	Place number
91-99	Brilliant level of functioning in a wide range of areas, never losing track of problems, sought out by others due to many positive qualities	
81-90	Good level of functioning in all areas, interested and engaged in a wide range of activities, socially functioning well, generally satisfied with life, only everyday problems and worries (e.g. an occasional argument with someone in the family)	
71-80	Insignificant, temporary, and situationally reduced social, occupational, or educational functioning (e.g. quite temporarily behind in schoolwork)	
61-70	Some difficulties with social, occupational, or educational functions (e.g. occasional truancy or raping at home).	
51-60	Moderate difficulties with social, work, or educational functions (very few friends, problems with those to cooperate with).	
41-50	Severe disruption of social, work, or educational functioning (e.g., no friends, unable to hold down a job, frequent shoplifting).	
31-40	Major functional impairment in several areas such as work, school, or family relationships (e.g. avoids contact with friends, neglects family, and cannot attend work	
21-30	Inability to function in almost all areas	
11-20	The person is occasionally unable to maintain minimal personal hygiene (e.g., smears stools).	
1-10	Permanently impaired ability to maintain minimal personal hygiene.	

9) Do you find the patient well-treated on current non-clozapine treatment?

☐ Yes ☐ No ☐ Don't know ☐ Other____

10) What reason(s) is there for this patient not being treated with clozapine?

Indicate the reason(s) for this patient not being treated with clozapine. Multiple concurrent reasons may be given. If a relevant reason is not represented among the possible answers below, please indicate the reason below point 10.l.

This patient is not being treated with clozapine because (tick):

☐ a. The patient's symptoms do not give rise to clozapine treatment

Elaborate on why:
____
____
____

☐ b. The patient has a somatic problem that contraindicates clozapine

State which somatic problem(s):
____
____
____

☐ c. The patient is being treated with other medications that contraindicate clozapine

Specify which medication:
____
____
____

☐ d. The side effects associated with clozapine treatment Indicate which possible side effects would particularly prevent you from offering clozapine to this patient:
____
____
____

Has the patient experienced one or more of these side effects with previously tested medication?

☐ Yes ☐ No ☐ Don't know

If yes, state which side effect(s) the patient has previously experienced:
____
____
____

☐ e. Because the patient is expected to refuse treatment with clozapine due to the requirement for blood sampling

☐ f. Because the patient is expected to refuse treatment with clozapine due to the side effect profile

 g. Because the patient is expected to refuse treatment with clozapine for another reason

State with which other reason you expect the refusal:
____
____
____

 h. Because the patient has refused treatment with clozapine

State when and with what reason the patient refused the treatment:
____
____
____

 i. Because the patient is expected to have poor adherence to the clozapine intake

 j. Because the patient is expected to have poor adherence to the blood sampling

 k. Because there are organizational conditions that make clozapine treatment difficult for this patient

State which circumstances:
____
____
____

 l. Other reason why this patient is not being treated with clozapine

State reason(s):
____
____
____

 m. Don't know.

Give reasons (e.g. I don't know the patient):
____
____
____

11) If multiple reasons are given under question 10, please state the most decisive reason:

 _. ____

12) Which (if any) of the measures below do you think could result in clozapine initiation for this patient? (Tick(s)):

☐ a. Offer to use local anesthetic ointment, prior to venous blood sampling

☐ b. Possibility of using finger prick tests for hematological monitoring, as an alternative to regular venous blood samples

☐ c. Longer intervals between blood tests

☐ d. Option to take blood tests and ECG in own outpatient clinic

☐ e. Option to take blood samples and ECG at the patient's home

☐ f. Access to information material developed especially for possible clozapine patients and their relatives (for example, a video with a patient story)

☐ g. Option to offer a meeting with a patient in established clozapine treatment (a "clozapine ambassador")

☐ h. Option to be able to refer to clozapine initiation via a specialized clozapine unit

☐ i. Possibility of using clozapine in other dispensing forms than tablets (e.g. oral drops or intramuscular injection (non-depot))

☐ j. Possibility of using clozapine as compulsory treatment

☐ k. Other measures that you believe would enable clozapine treatment for this patient:
____
____
____

☐ l. No measures are relevant for this patient

Why not?
____
____
____

☐ m. Don't know.

Give reason:
____
____
____

13) If several measures are selected under question 13, please indicate the most relevant measure for this patient:

☐ _. ____

14) Any explanatory comments?:
____
____
____
____
____
____
____
____
____

15) Where do you work?

☐ Inpatient section ☐ District outpatient psychiatry ☐ Other_____________________

Many thanks for your help.

If further clarification of the subject is needed, for example in the form of an in-depth interview, will you agree to be contacted?

☐ Yes

☐ No

Date of completing the questionnaire: ____________

The completed questionnaire should be returned to the following e-mail address: xxxx
