# Supplementary material for: Non-prescribing of clozapine for outpatients with schizophrenia in real-world settings: The clinicians’ perspectives
Source: Schizophrenia (Heidelb). 2023 Dec 22;9(1):91. doi: 10.1038/s41537-023-00423-3 (PMC10746712; doi:10.1038/s41537-023-00423-3)
Supplement: Supplementary file 2 — S2 [file 41537_2023_423_MOESM2_ESM.pdf]

Interview guide

Non-prescribing of clozapine for outpatients with schizophrenia in real-world settings: The clinicians' perspectives

The objective of this interview is to obtain individual psychiatrists' perspectives and elaborations on the survey results regarding non-prescribing of clozapine for selected patients.

The interview has 3 parts:

Part 1 - introduction with exploration of the psychiatrists' experience with and general attitude towards clozapine treatment,

Part 2 – elaborations on the main result regarding reasons for non-clozapine treatment,

Part 3 – elaborations on the main results regarding clozapine-facilitating initiatives.

The interview will last approximately 30 minutes.

Part 1

I know from the survey questionnaire that you have experience with clozapine treatment and that you have commenced patients in clozapine treatment yourself. Now, I would like to hear what your general experiences with clozapine treatment are?

Probes:

- What do you think of clozapine treatment?
- How do you find the effect?
- Who would be eligible for clozapine treatment?
- If you were to describe a clozapine-eligible patient with a CGI and GAF score, in what range would they score? (show the actual scales)
- What do you think is the most frequent, decisive reason why otherwise clozapine-eligible patients are not initiated on clozapine treatment?
- What do you think it would take if more patients were to be initiated on clozapine treatment?

Part 2

In the survey, questionnaires were sent to psychiatrists and clinical care providers responsible for the treatment of selected patients; patients who, based on their history of medications, appeared to be eligible for clozapine treatment.

You were asked to rate the patients symptom severity, level of functioning, whether you found them well-treated or not and to answer why they were not treated with clozapine.

Questionnaires regarding 39 patients were returned. The median CGI rating of patients was 5.0 (5.0-6.3) and the median GAF score was 33.0 (25.0-42.1) (show on printed scales).

27 of the 39 patients were rated well-treated by one or more clinicians (there were no differences between well-treated and not well-treated patients in terms of CGI and GAF scores).

- What do you think about that?
- When is a patient well-treated in that context?

Psychiatrists and care providers agreed quite well in their clinical ratings, but when it came to whether the patients were considered well-treated or not you only agreed in approximately 1/3 cases. In most cases, it was the psychiatrists who found the patients well-treated.

- What do you think about that?

In the questionnaires, you could choose between 13 different reasons why the patient was not in clozapine treatment (show the list with reasons).
You could choose several simultaneous reasons, you could add reasons yourself, and you were also asked to choose the most important reason/the main reason.

The most frequently chosen reason was related to hematological monitoring – especially the expected non-compliance with blood sampling was chosen often (show in list).
However, the psychiatrists' most frequently chosen main reason was that the patient was well-treated (show on the list).
Not a single psychiatrist chose hematological monitoring as the main reason.

- What do you think about that?

Probe

- What does it mean to be too well-treated for clozapine treatment?

In contrast, the care providers' most frequently chosen main reason was the hematological monitoring.

- What do you think about that?

In some questionnaires there was a comment stating that the patient was too ill for clozapine treatment.

- What does it mean to be too ill for clozapine treatment?

Part 3

In the survey questionnaires, you could also choose between 13 different answers regarding facilitators of clozapine treatment for the patient in question (show the list with facilitating initiatives).

You could choose several simultaneous initiatives, you could add initiatives yourself, and then you were asked to choose the most relevant facilitator.

- What do you think about the proposed initiatives on the list?
  (show the list of facilitating initiatives)

The most frequently chosen answer was that no initiatives were relevant – often accompanied by a note stating that the patient was too ill for clozapine treatment or as well-treated as possible.
This answer was also the psychiatrists' most frequently chosen main answer

- What do you think about that?

The most frequently mentioned facilitating initiatives in the literature so far have been related to blood sampling - especially the implementation of Point of Care devices for finger-prick blood sampling instead of venous blood sampling.

In the questionnaires, the psychiatrists chose initiatives aiming at blood sampling as the most relevant initiative in very few cases only (2/27 cases)

- What do you think about that?

In comparison, the care providers did choose an initiative related to blood sampling as the most relevant one in most cases.

- Why do you think that psychiatrists' and care providers' perspectives on main barriers and facilitators seem to differ?

- Is there anything you would like to add?

Thank you for your time.
